# Disagreements Within the US Food and Drug Administration Regarding Approval of Novel Therapeutic Agents, 2011-2015

**DOI:** 10.1001/jamanetworkopen.2020.9498

**Published:** 2020-07-24

**Authors:** Andrea MacGregor, Audrey D. Zhang, Joshua D. Wallach, Joseph S. Ross, Matthew Herder

**Affiliations:** 1 Schulich School of Law, Dalhousie University, Halifax, Nova Scotia, Canada; 2 New York University School of Medicine, New York, New York; 3 Department of Environmental Health Sciences, Yale School of Public Health, New Haven, Connecticut; 4 Department of Internal Medicine, Yale School of Medicine, New Haven, Connecticut; 5 Health Law Institute, Schulich School of Law, Dalhousie University, Halifax, Nova Scotia, Canada; 6 Department of Pharmacology, Faculty of Medicine, Dalhousie University, Halifax, Nova Scotia, Canada

## Abstract

This cross-sectional study examines the frequency of disagreements within the US Food and Drug Administration (FDA) regarding approval of novel therapeutic agents.

## Introduction

Thirty days after a novel therapeutic agent, a new molecular entity, or original biologic is approved, the US Food and Drug Administration (FDA) must publicly disclose its approval package, including scientific reviews completed by FDA disciplines (eg, pharmacology, statistical, and medical reviewers) and any available assessments by agency leadership.^1^ Although reports of internal disagreement have surfaced,^2^ it is unclear how often such disagreements occur. Disagreements document differing points of view or engaged discussion and may, thus, capture important scientific debates or signal challenging decisions within the agency. We sought to determine the frequency of disagreements within the FDA regarding approval of novel therapeutic agents.

## Methods

This cross-sectional study did not require institutional review board approval or patient informed consent because it was based on publicly available information and involved no patient records, in accordance with 45 CFR §46. This study follows the Strengthening the Reporting of Observational Studies in Epidemiology (STROBE) reporting guideline for cross-sectional studies.

Between May and September 2019, we identified all approval packages for novel therapeutic agents approved by the FDA from January 2011 to December 2015 using the Drugs@FDA database. Disagreements were defined as instances where multiple reviewers and/or leadership (whether part of the same discipline or not) disagreed about approving a drug, the indicated patient population (eg, patient age), and/or the parameters of the drug’s approval (eg, postmarketing requirements). We searched for disagreements in 2 ways. First, we reviewed the Summary Review, the Office Director Memo, the Cross-Disciplinary Review, and the Medical Review, which tend to describe the recommendations and disagreements. Second, we used key word searches to identify disagreements located elsewhere in the package. The data were extracted by 1 author (A.M.); uncertain cases were resolved through discussion with 2 or 3 other authors (M.H., J.D.W., and A.D.Z.). The frequency of disagreements within and between different disciplines and/or FDA leadership (eg, Division Director) was recorded and tabulated using Excel software version 16.31 (Microsoft Corp). Data analysis was performed from June to November 2019.

## Results

From 2011 through 2015, the FDA published 174 approval packages for novel therapeutic agents (Table 1). The most common therapeutic areas were cancer (46 agents [26.4%]) and infectious diseases (27 agents [15.5%]); 72 agents (41.4%) were first in class, and the FDA was the first major regulatory agency to approve the drug for 118 agents (67.8%).

**Table 1.** Novel Therapeutic Agents Approved 2011-2015

Year, agent		
2011 (n = 29)		
Aflibercept^a^ Asparaginase erwinia chrysanthemi Ruxolitinib phosphate Clobazam Deferiprone Crizotinib Icatibant acetate^b^^,^^c^ Brentuximab vedotin Vemurafenib	Ticagrelor^b^ Indacaterol maleate Rivaroxaban Belatacept Ezogabine Fidaxomicin Telaprevir Rilpivirine hydrochloride Boceprevir Linagliptin	Abiraterone acetate Gabapentin enacarbil Vandetanib Ipilimumab Belimumab Roflumilast Azilsartan kamedoxomil^a^ Vilazodone hydrochloride^c^ Spinosad
2012 (n = 36)		
Crofelemerb^b^ Apixaban^c^ Bedaquiline fumarate Lomitapide mesylate Teduglutide recombinant Pasireotide diaspartate^a^ Ponatinib hydrochloride Raxibacumab Cabozantinib s-malate Tofacitinib citrate Omacetaxine mepesuccinate^c^ Perampanel^c^^,^^d^	Ocriplasmin Regorafenib Teriflunomide^a^^,^^c^ Bosutinib monohydrate^a^^,^^c^^,^^d^ Enzalutamide Linaclotide^a^^,^^c^^,^^d^ Tbo-filgrastim Cobicistat; elvitegravir; emtricitabine; tenofovir disoproxil fumarate Ziv-aflibercept Aclidinium bromide Carfilzomib	Mirabegron Lorcaserin hydrochloride Pertuzumab^b^ Taliglucerase alfa Avanafil Peginesatide acetate Lucinactant Tafluprost Ivacaftor Vismodegib^c^ Axitinib Ingenol mebutate Glucarpidase
2013 (n = 24)		
Umeclidinium bromide; vilanterol trifenatate Sofosbuvir Simeprevir sodium Luliconazole Ibrutinib Eslicarbazepine acetate^b^ Obinutuzumab Macitentan Riociguat^a^	Bazedoxifene acetate; estrogens, conjugated Vortioxetine hydrobromide Dolutegravir sodium Afatinib dimaleate Dabrafenib mesylate Trametinib dimethyl sulfoxide Radium Ra-223 dichloride	Fluticasone furoate; vilanterol trifenatate Canagliflozin^a^^,^^d^ Dimethyl fumarate^b^ Ospemifene Ado-trastuzumab emtansine Pomalidomide Mipomersen sodium Alogliptin benzoate^c^
2014 (n = 38)		
Nivolumab Ceftolozane sulfate; tazobactam sodium Dasabuvir sodium; ombitasvir; paritaprevir; ritonavir Olaparib^a^ Peramivir Finafloxacin Blinatumomab Nintedanib esylate Pirfenidone Ledipasvir; sofosbuvir Netupitant; palonosetron hydrochloride	Dulaglutide^a^ Naloxegol oxalate Pembrolizumab Eliglustat tartrate Peginterferon beta-1a Suvorexant Oritavancin diphosphate Empagliflozin Olodaterol hydrochloride Idelalisib Tavaborole Belinostat Tedizolid phosphate Dalbavancin hydrochloride^a^^,^^b^	Vedolizumab^d^ Vorapaxar sulfate^c^ Ceritinib Siltuximab Ramucirumab^c^ Albiglutide Apremilast^c^ Miltefosine^c^ Metreleptin Droxidopa^a^^,^^b^ Elosulfase alfa Tasimelteon Dapagliflozin
2015 (n = 48)		
Lesinurad^b^^,^^c^ Selexipag^c^ Sugammadex sodium Alectinib hydrochloride Sebelipase alfa Elotuzumab Necitumumab Ixazomib citrate Daratumumab Osimertinib mesylate Cobimetinib fumarate Cobicistat; elvitegravir; emtricitabine; tenofovir alafenamide fumarate Mepolizumab^c^^,^^d^ Asfotase alfa Trabectedin Patiromer sorbitex calcium	Idarucizumab^c^ Aripiprazole lauroxil Insulin aspart; insulin degludec Insulin degludec Tipiracil hydrochloride; trifluridine Cariprazine hydrochloride Uridine triacetate Rolapitant hydrochloride Evolocumab Flibanserin^b^ Alirocumab^c^^,^^d^ Daclatasvir dihydrochloride Sonidegib phosphate Brexpiprazole Sacubitril; valsartan^a^ Ivacaftor; lumacaftor	Cangrelor^a^^,^^b^ Eluxadoline Olodaterol hydrochloride; tiotropium bromide Deoxycholic acid Ivabradine hydrochloride^a^^,^^d^ Cholic acid^d^ Dinutuximab Filgrastim-sndz Isavuconazonium sulfate Avibactam sodium; ceftazidime Panobinostat lactate^b^ Lenvatinib mesylate Palbociclib Parathyroid hormone Secukinumab^a^^,^^c^ Edoxaban tosylate^d^

^a^ Denotes approval packages containing 1 or more disagreements pertaining to some other form of disagreement not mentioned in the subsequent footnotes.

^b^ Denotes approval packages containing 1 or more disagreements regarding approval.

^c^ Denotes approval packages containing 1 or more disagreements pertaining to parameters of approval.

^d^ Denotes approval packages containing 1 or more disagreements pertaining to population or indication.

Forty-two (24.1%) approval packages contained at least 1 disagreement: 12 (6.9%) included a disagreement about whether to approve a drug, 10 (5.7%) disagreed over the patient population for which the drug was indicated, and 35 (20.1%) disagreed regarding the parameters of approval, including 20 about postmarketing requirements, safety warnings, or risk evaluation and management strategies, and 15 about other issues, such as drug label phrasing.

Of 155 instances of disagreement, 18 (11.6%) were among reviewers within the same discipline, whereas 137 (88.4%) occurred between different disciplines and/or leadership. The most frequently involved parties were medical reviewers (27 cases [17.4%]), members of agency leadership (eg, the Division Director; 33 cases [21.3%]), the Cross-Discipline Team Lead (23 cases [14.8%), and the Office Director (19 cases [12.3%]) (Table 2). Among the 12 disagreements regarding approval, 11 were approved with a postmarketing requirement or risk evaluation and management strategy.

**Table 2.** US Food and Drug Administration Disagreements Over New Drug Approvals, Populations Indicated, and the Parameters of Approval by Subject, Leadership, and Discipline (Total Instances)^**a**^

Variable	Approvals (dissents), No.^b^	Population or indication, No.	Approval parameters, No.	Other, No.	Total by discipline, No. (%) (n = 155)
Agency leadership					
Division director	9 (1)	8	10	6	33 (21.3)
Office director	5 (0)	2	8	4	19 (12.3)
Cross-disciplinary team leader	8 (4)	5	6	4	23 (14.8)
Agency disciplines					
Medical	6 (5)	6	8	7	27 (17.4)
Clinical pharmacology	2 (1)	2	3	5	12 (7.7)
Statistics	4 (3)	4	1	3	12 (7.7)
Safety	1 (1)	5	5	1	12 (7.7)
Pediatric	0 (0)	1	3	1	5 (3.2)
Chemistry	4 (1)	0	0	0	4 (2.6)
Nonclinical pharmacology	1 (0)	0	2	1	4 (2.6)
Office of Scientific Investigations and other reviewers of regulatory issues	1 (0)	0	1	0	2 (1.3)
Division of Risk Management and other reviewers of the Risk Evaluation and Mitigation Strategy	0 (0)	0	2	0	2 (1.3)
Microbiology	0 (0)	0	0	0	0 (0.0)
Total by subject (n = 155)	41	33	49	32	

^a^ In 8 cases, the other party disagreeing with the disciplines listed here was not a member of 1 of the standard disciplines or leadership that are listed here. For example, in the case of the novel therapeutic agent sacubitril-valsartan (Entresto), a reviewer filed a nonstandard unsolicited review containing conclusions with which members of the standard disciplines disagreed.

^b^ The number in parentheses reflects the number of disagreements in which the discipline (or leadership) was the party recommending against approval. These numbers do not include within-discipline dissents, in which 1 reviewer recommended against approval but the discipline as a whole ultimately supported approval.

## Discussion

Among all approval packages for novel therapeutics approved by the FDA from 2011 to 2015, disagreements were common over new drug approvals, populations indicated, and the specific parameters of the approval. Given the complexity of determining drug safety and efficacy and the challenge of extrapolating to broad populations from a limited number of small, narrowly defined clinical trials,^3^ disagreements within the FDA are not surprising and likely represent differing points of view that may inform pharmacovigilance efforts, as well as public discourse.^4^

Our study was limited to disagreements recorded within approval packages. Where disagreements may be unrecorded, our analysis may underestimate their prevalence. We also did not assess whether disagreements were discussed by advisory committees or associated with particular outcomes (eg, safety warnings discovered after approval).

Nevertheless, our findings have important implications for the FDA’s recent move to publish only integrated reviews in lieu of reviews by each discipline and agency leadership.^5^ It raises questions about whether disagreements within the agency will continue to be published in compliance with the law.^1,6^
